# Japanese Consumers’ Attitudes towards Obtaining and Sharing Health Information Regarding Over-the-Counter Medication: Designing an Over-the-Counter Electronic Health Record

**DOI:** 10.3390/healthcare11081166

**Published:** 2023-04-18

**Authors:** Guyue Tang, Kairi Izumi, Megumi Izumisawa, Shinichi Koyama

**Affiliations:** 1 Graduate School of Comprehensive Human Sciences, University of Tsukuba, Ibaraki 305-8577, Japan; tangguyue@outlook.com (G.T.);; 2 Department of Pharmacy, Nihon University, Chiba 274-0063, Japan; 3 Faculty of Art and Design, University of Tsukuba, Ibaraki 305-8574, Japan

**Keywords:** electronic health record, self-medication, digital health, eHealth literacy, OTC medication, health management

## Abstract

Designing an over-the-counter medication electronic health record (OTC-EHR) may help improve OTC usage. An online survey for the conceptual OTC-EHR design examined participant characteristics, attitudes towards obtaining user-shared OTC medication information, health-related application usage, and the inclination to share anonymized health information. Descriptive statistics, tests to assess statistical significance, and text mining were used to analyze the results. The findings revealed that Japanese consumers, particularly those with high eHealth literacy and women, possess relatively positive attitudes towards obtaining user-shared OTC medication information than those with low eHealth literacy (*t* (280.71) = −4.11, *p* < 0.001) and men (*t* (262.26) = −2.78, *p* = 0.006), respectively. Most consumers own smartphones but do not use health-related applications. A minority held positive attitudes about sharing anonymized health information. The perceived helpfulness of OTC-EHR was positively associated with the usage of health-related applications (χ^2^ (4) = 18.35, *p* = 0.001) and attitude towards sharing anonymized health information (χ^2^ (3) = 19.78, *p* < 0.001). The study findings contribute towards OTC-EHR’s design to enhance consumers’ self-medication and reduce risks, while the psychological barriers to sharing anonymized health information should be improved by increasing the OTC-EHR’s penetration rate and engaging in appropriate information design.

## 1. Introduction

Self-medication practices have increased in Japan since the revised Pharmaceutical Affairs Act was enacted in 2009 [1]. As medicines that can be purchased without a prescription, over-the-counter (OTC) medication plays an important role in self-medication [2,3,4,5,6,7]. Consumers make decisions for self-medication based on the information published by manufacturers and the additional information from other sources. Obtaining inappropriate information about OTC medication presents potential barriers and health risks for self-medication [8,9,10]. On the one hand, manufacturers gather factual medical information before launching a medication and subsequently releasing it to the public. However, previous studies have reported potential barriers and risks in obtaining information for consumers from packaging and labels [11,12,13,14,15]. On the other hand, obtaining medical information in addition to what is provided by manufacturers also poses potential barriers and risks, especially when such information is obtained through the Internet [16,17,18,19]. As a country promoting self-health management, health information technology (HIT) plays an active role in Japan [20,21]. During the COVID-19 pandemic, Japanese consumers were able to use health-related applications to self-monitor their physical condition and record their medication information on smartphones. Considering the positive roles of mobile Internet, health-related applications on smartphones, and the advantages of information processing in HIT [22,23,24,25], we assume that an OTC medication electronic health records (OTC-EHR) design based on obtaining and sharing health information in relation to self-medication may improve potential health risks caused by inappropriate information acquisition. This study attempts to investigate consumers’ attitudes towards obtaining and sharing health information and attitudes towards health-related applications and their characteristics to test the hypotheses regarding the conceptual design of the OTC-EHR.

The electronic health record (EHR) is a well-established concept in the field of HIT, with more positive characteristics than negative ones [26]. It actively contributes to patient-physician communication [22,27] and helps serve public health [26]. Studies have shown that co-interventions involving EHR and nurses’ assistance positively impacted medication self-management [28,29], and improvements in EHR design are required for effective self-management [30]. Due to the positive impacts of EHR, it has been widely used in the clinical field, but it is usually overlooked for non-prescription medicines [31,32,33]. OTC medication plays an essential role in Japan [1,20]. Researchers in Japan have investigated the potential positive effects of EHR, and most respondents have a positive attitude towards it. However, studies on EHR are limited in Japan [21,34,35]. As for OTC-EHR, related research is required to be continued [31], especially from the consumers’ perspective.

Regarding Japanese consumers’ attitudes towards OTC-EHR, considering their favorable attitudes towards technology may facilitate a greater use of OTC-EHR.

Moreover, the perceived usefulness of EHR may positively affect EHR use [27,36]. Considering the abovementioned information, the following hypotheses were formulated:

**H1.**  *Consumers exhibit a positive attitude towards obtaining user-shared health information on OTC medication*.

**H2.**  *Consumers exhibit a positive attitude towards sharing anonymized health information while using OTC medication*.

**H3.**  *There is a positive association between consumers obtaining and sharing health information regarding OTC medication*.

Moreover, since the conceptual design of OTC-EHR will be based on the medication information system design, it is necessary to consider the influence of consumers’ experience with related health applications on their attitude towards OTC-EHR use.

**Figure 1:**
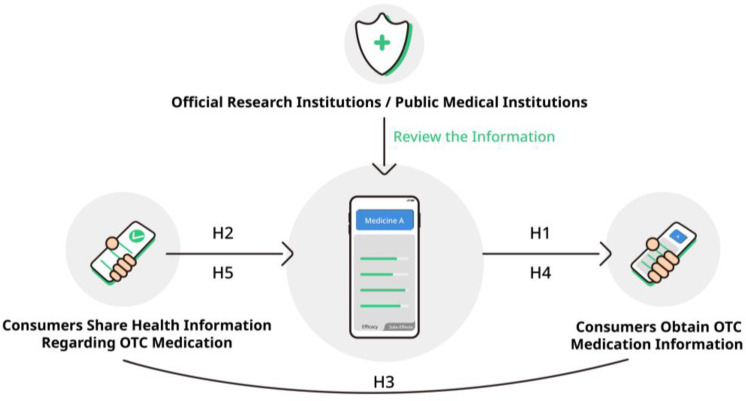
The conceptual design speculative model for OTC-EHR.

During the COVID-19 pandemic, in Japan, people could record their health status and report it through smartphone applications [37,38], thereby encouraging them to use HIT for self-health management. Examples include the medication notebook application and the health observation application. Users can use the medication notebook application to record the name of the medicine and usage, along with past experiences of allergies and side effects [37]. The health observation application is a kind of physical condition observation application, which was widely used in Japan to record one’s physical condition during the COVID-19 pandemic [38]. Considering the potential positive and negative factors of OTC-EHR [31], and based on the kind of database of medication notebook and health observation application that records and reports personal health information [37,38], this study proposes a consumer-centered OTC-EHR conceptual design based on obtaining and sharing OTC medication information with official research institutions and public medical institutions as review agencies [39]. Figure 1 shows the conceptual design speculative model for the OTC-EHR. Specifically, consumers share health information while using the OTC medication, called user-shared health information, which the review agencies review and make accessible to the users of the OTC-EHR. This design is expected to improve the potential risks facing consumers obtaining additional OTC medication information that has not been authenticated for use in self-medication. Since this study focuses on consumer attitudes toward obtaining and sharing information in the OTC-EHR, how agencies review relevant information will not be discussed in the present study.

In addition, consumer behavior regarding self-medication and HIT may vary according to eHealth literacy, gender and age. eHealth literacy is the consumer’s capacity to use digital health information to address health-related issues and is essential in digital healthcare [8,10,40]. Previous studies suggest eHealth literacy’s positive role in Japanese consumers’ related health behaviors and digital health information acquisition [39,41]. Other studies have suggested that women may have a stronger motivation to seek Internet health information. Although younger adults use the Internet more overall, middle-aged adults are more likely to seek health information online [39,42,43,44]. Moreover, regarding OTC medication purchases, women and younger adults may buy OTC medicines more frequently [1,7].

We therefore propose the following hypotheses considering eHealth literacy and consumers’ attitudes:

**H4.**  *Consumers with high eHealth literacy have a greater inclination to obtain user-shared information regarding OTC medication*.

**H5.**  *Consumers with high eHealth literacy are more optimistic about sharing anonymized health information regarding OTC medication*.

The present study aims to investigate consumers’ relevant attitudes to test the research hypotheses for the OTC-EHR conceptual design from two perspectives: (i) attitudes towards obtaining user-shared OTC medication information; and (ii) the usage of health-related applications and the inclination to share anonymized health information.

## 2. Materials and Methods

### 2.1. Participants

A total of 450 participants from the Greater Tokyo Area of Japan attended a survey in February 2022 through Freeasy, a leading online survey platform in Japan. Participants received points that could be used instead of money at certain stores as a reward [45,46]. Participants were categorized into the following age groups: 20–29 years group (*N* = 150); 30–39 years group (*N* = 150); and 40–49 years group (*N* = 150). Of the participants, 50% (255) were men. Considering the potential influence of medical knowledge, this recruitment excluded respondents engaged in medicine-related occupations [11]. Freeasy recruited participants from registered panels who were willing to participate in the survey and earn points, and the response rate was 100%. We referred to the age groups of participants from previous studies and considered potential Internet-based device usage experiences [1,41,47]. We considered an online survey to be appropriate for this study because the participants could use the Internet. Moreover, the Greater Tokyo Area is one of the leading regions in Japan’s economic and technological development; therefore, conducting a survey regarding the OTC-EHR here is representative.

To exclude random and implausibly fast responses, this study used a screening question and the response time taken to complete the survey as evaluation criteria. The following screening question was used: “Which of the following is not mentioned in this survey? (a) How to purchase medicines; (b) Methods of collecting medical information; (c) Annual consumption of medicines; (d) Medication guidance by pharmacists”. Those who chose option (c) were excluded. In terms of the response time, we calculated the time taken to submit the survey without checking the contents appropriately as around 1 min and 40 s and used this as an additional criterion. The implausibly fast responses were excluded. Finally, the data of 288 responses were analyzed.

We calculated the effect size and power through G*Power, which suggested that 288 responses in this study would be sensitive to the effect size of 0.33 with 80% power (alpha = 0.05, two-tailed). The actual powers in this study for eHealth literacy and gender differences were 0.99 and 0.80, respectively, confirming a reasonably sized sample [48,49,50]. In addition, the corresponding actual effect size for other statistical methods was reported.

### 2.2. Measures

The online survey comprised four sections: (i) survey introduction; (ii) participant characteristics including eHealth literacy, gender, and age. eHealth literacy was measured using J-eHEALS [51], which is the Japanese version of the eHealth Literacy Scale [52]; (iii) attitudes towards obtaining user-shared OTC medication information; (iv) the usage of health-related applications and the inclination to share anonymized health information. (See Supplementary Table S1 for a list of survey questions and hypotheses).

#### 2.2.1. Survey Introduction

We introduced the participants to what OTC medication is and informed them about the purpose of this anonymous survey, research ethics, research institutions, and the researchers in charge. Participants could withdraw from this survey at any time. After that, they were asked to comply with the following precautions while answering the survey: (i) answer alone, without consulting anyone else; (ii) do not eat or drink while answering the survey; (iii) answer it in a quiet room without music, TV, etc.

#### 2.2.2. Participant Characteristics

Participant characteristics including eHealth literacy, gender, and age were examined. Participants’ eHealth literacy was measured using J-eHEALS [51]. Responses to questions on eHealth literacy were provided on a five-point Likert scale ranging from “strongly agree = 5” to “strongly disagree = 1”. A higher score indicated better perceived eHealth literacy. The Cronbach’s alpha value of J-eHEALS was 0.93 in the present study.

#### 2.2.3. Attitudes towards Obtaining User-Shared OTC Medication Information

This study used three questions to examine consumer attitudes towards obtaining user-shared OTC medication information. The first question was the following: (i) If there were a database of post-dose health information shared from past users of the OTC medication you purchase, do you think it would be helpful when deciding to choose the medication? The response options ranged from “very helpful = 5” to “not helpful at all = 1”. The next question was as follows: (ii) What information would you like to refer to in this user-shared report? Respondents were asked to select all suitable answers from the following options: “efficacy of medicines”, “safety of medicines”, “average time from taking a medicine to effects”, “duration of drug effect”, “symptoms of side effects”, “incidence of side effects”, “others”, and “I do not want to know any information”. The third question was the following: (iii) Enter the reasons for choosing the responses in the free response text box. These questions were used to test hypotheses H1 and H3 pertaining to attitudes on obtaining health information.

#### 2.2.4. Usage of Health-Related Applications and Inclination to Share Anonymized Health Information

Two questions were used to examine the usage of health-related applications and attitudes towards sharing anonymized health information. The first question was the following: (i) Do you use a medication notebook application or an application that monitors your health condition, such as entering body temperature, on your smartphone? Respondents were asked to select one of the following answers that best applies to them: “I only use the medication notebook application”, “I only use the health observation application”, “I use both the medication notebook and the health observation applications”, “I have a smartphone but use neither application”, and “I do not use a smartphone”. The following question was then asked: (ii) Do you think it is acceptable for information about your physical condition and medicines used entered into the application to be provided to others after anonymization? The answers were “I think it is a good thing”, “not okay”, “neither”, and “I do not know”. These questions aimed to test hypotheses H2 and H4 on sharing of information.

### 2.3. Statistical Analyses

The descriptive statistics characteristics of the respondents were summarized, including the frequency and percentage for categorical variables and the mean and standard deviation for continuous variables. For J-eHEALS scores, participants were divided into two categories (high or low literacy) relative to the median group value (median 23.00, inter-quartile range 18.00–27.00) based on previous studies [41,53,54,55].

The chi-square, *t*-test, and one-way ANOVA were used to examine the differences based on eHealth literacy, gender, and age. SPSS 28.0 was used to perform the statistical analysis, and *p* values less than 0.05 were considered statistically significant. The effect sizes for the Chi-square goodness of fit, Chi-square crosstab, *t*-test, and ANOVA were reported as Cohen’s *W*, Cramér’s *V*, Cohen’s *d*, and *η^2^*, respectively [56,57,58].

Moreover, the text-mining method was additionally performed in this study for free response text using the KH Coder [59,60], which helps researchers keep track of the most frequently used phrases, identify word associations, and group words into logical clusters [61,62]. Before formally analyzing the text based on the original sentences, we used KH Coder’s programming function to merge close synonyms to obtain more accurate analysis results. For example, the different expressions for the effectiveness and side effects of medication in Japanese were converted into unified “effectiveness” and “side effects” clusters.

## 3. Results

### 3.1. Participant Characteristics

The participants were categorized into the following age groups: 32.99% (95) were in the 20–29 years group (mean = 24.81, SD = 2.90); 30.55% (88) in the 30–39 years group (mean = 32.94, SD = 2.94); and 36.46% (105) in the 40–49 years group (mean = 44.86, SD = 2.93). Moreover, 47.92% (138) were men. In terms of eHealth literacy, the mean score on J-eHEALS was 22.65 ± 6.64.

### 3.2. Attitudes towards Obtaining User-Shared OTC Medication Information

Table 1 presents the attitudes towards obtaining user-shared health information during OTC medication use (χ^2^ (4) = 219.33, *p* < 0.001, *W* = 0.87). The average score of perceived helpfulness was 3.51 (SD = 0.85). One sample *t*-test implied that the user-shared health information was perceived as relatively more helpful compared to “neither” (*t* (287) = 10.30, *p* < 0.001, d = 0. 61).

**Figure 2:**
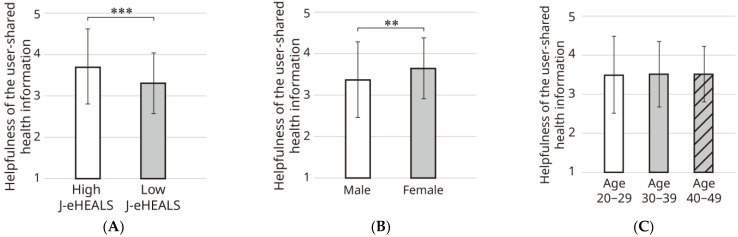
Perceived helpfulness of obtaining user-shared health information on OTC medication. (**A**) Helpfulness of the user-shared health information based on different levels of eHealth literacy (High J-eHEALS: mean = 3.72, SD = 0.73; Low J-eHEALS: mean = 3.32, SD = 0.90); (**B**) helpfulness of the user-shared health information based on gender (Men: mean = 3.37, SD = 0.93; Women: mean = 3.65, SD = 0.74); (**C**) helpfulness of the user-shared health information based on age groups (20–29 years: mean = 3.49, SD = 0.99; 30–39 years: mean = 3.52, SD = 0.84; 40–49 years: mean = 3.52, SD = 0.71). ** *p* < 0.01, *** *p* < 0.001.

More specifically, as Figure 2 shows, an independent *t*-test implied that consumers with high eHealth literacy tended to have more positive attitudes towards user-shared health information regarding OTC medication (*t* (280.71) = −4.11, *p* < 0.001, *d* = 0.49). Women displayed more positive attitudes than men (*t* (262.26) = −2.78, *p* = 0.006, *d* = 0.33). The one-way ANOVA showed no significant difference among the age groups (*F* (2, 285) = 0.04, *p* = 0.97, *η*^2^ < 0.001).

Table 2 presents the results pertaining to the information respondents wished to obtain. The chi-square test (excluding the respondent who chose “others” and entered specific information) implied that women had slightly broader information needs from user-shared information than did men (χ^2^ (6) = 14.29, *p* = 0.03, *V* = 0.12), such as side symptoms, time to produce the effect, duration of the medication, and side effect incidence rate. No significant differences were found based on different levels of eHealth literacy (χ^2^ (6) = 4.54, *p* = 0.60, *V* = 0.07) and age groups (χ^2^ (12) = 4.95, *p* = 0.96, *V* = 0.05). In addition, two respondents chose “others” and answered that they wanted to obtain the following information: “Pre-existing medical conditions and the general health condition of the person using the medicines and any other medicines or combinations of medications” and “Users’ personal information”.

**Figure 3:**
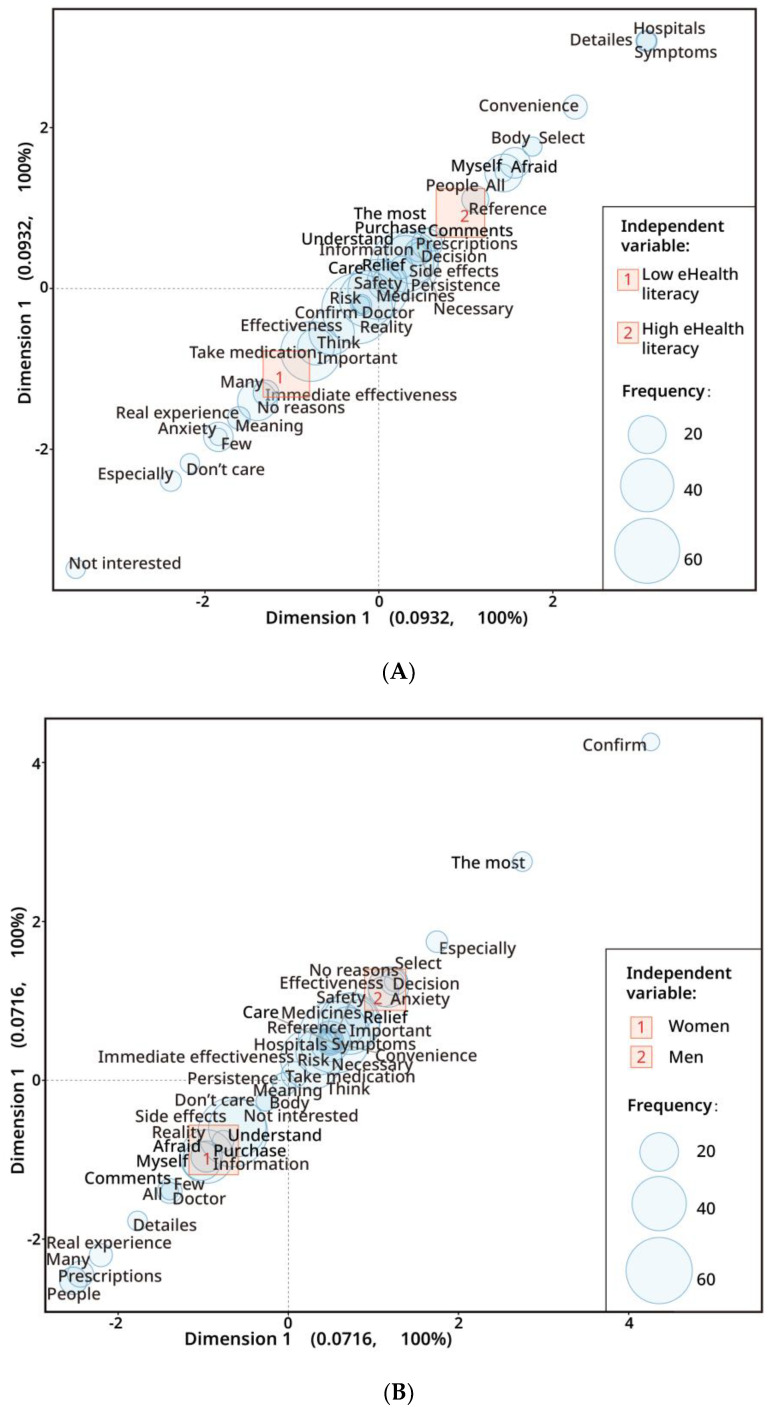

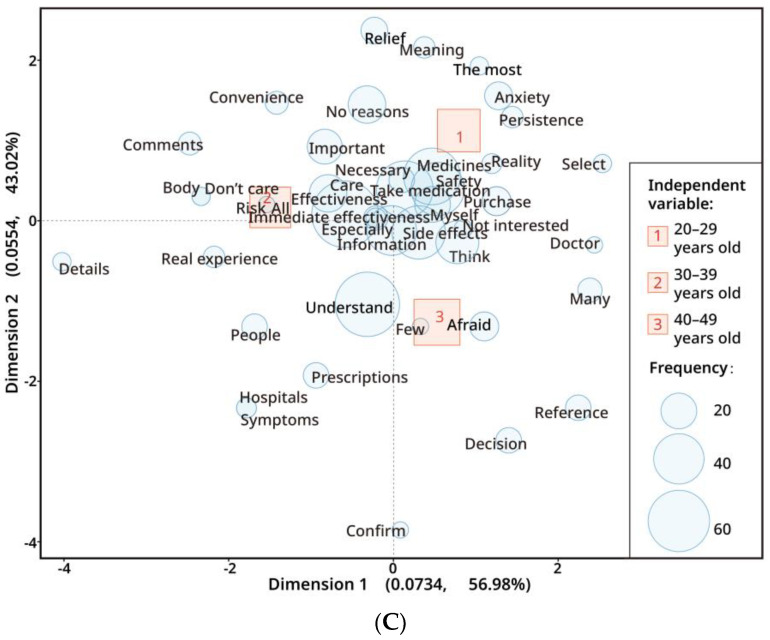
The correspondence analysis of the reasons refers to user-shared OTC medication information. (**A**) Analysis based on different eHealth literacy levels; (**B**) analysis based on gender differences; (**C**) analysis based on different age groups. In this plot, uncharacteristic words uniformly found in all independent variables are plotted near the origin (0,0); the shorter the distance between a word and independent variables, the more specific the association; the further the distance from the origin, the more characteristic the word and the more distinguishable it is from other independent variables.

To identify the reasons for choosing the information, we performed the text-mining analysis of the free responses, as Figure 3 shows. Most participants expressed concerns about comprehending information about physical safety, side effects, efficacy, persistence, etc., from the health information, regardless of eHealth literacy levels, gender, or age. More specifically, some observations were noted. First, some consumers with high eHealth literacy expressed that they wanted to know more about attention to specific symptoms, their physical condition, and whether the user went to the hospital. In contrast, some of the respondents with lower eHealth literacy expressed anxiety and indifference to medication information. Second, compared to men, some women expressed that they would like more detailed information and information on comparison with prescription medicines, the doctor’s opinion, other people’s real experiences, etc. In contrast to men, women mentioned side effects more frequently. Men talked about effectiveness more often than did women, and some men expressed anxiety about medications. Third, the answers of a few respondents in the 20–29 years group respondents expressed the need to alleviate anxiety about medication and concerns about medication persistence through user-shared information; however, other respondents in this group did not provide reasons. A small number of respondents in the 30–39 years group expressed concern about their physical condition, comments from others, and real experiences, particularly the details of medication information. Few respondents in the 40–49 age group mentioned making decisions by reference. Participants in the 30–39 and 40–49 years groups expressed more concerns about symptoms, hospitals, and prescription medicines than did those in the 20–29 years group.

### 3.3. Usage of Health-Related Applications and the Inclination to Share Anonymized Health Information

Regarding the results of health-related application usage, as Table 3 shows, more than 18% (53) of respondents used at least one application. Most consumers used smartphones only (χ^2^ (4) = 445.68, *p* < 0.001, *W* = 1.24). The percentage of health-related applications use for consumers with high eHealth literacy was slightly more heightened than those with low eHealth literacy (χ^2^ (4) = 10.34, *p* = 0.03, *V* = 0.19). The proportion of participants with low eHealth literacy who use the health observation application or both applications is lower than those with high eHealth literacy. No significant difference was found between genders (χ^2^ (4) = 8.49, *V* = 0.17, *p* = 0.08) or age brackets (χ^2^ (8) = 9.82, *p* = 0.28, *V* = 0.13).

Table 4 shows the results of sharing anonymized health information with others (χ^2^ (3) = 30.58, *p* < 0.001, *W* = 0.33). Significant differences were implied between consumers with different eHealth literacy levels (χ^2^ (3) = 8.83, *p* = 0.03, *V* = 0.18). Significant differences were found between genders (χ^2^ (3) = 9.22, *p* = 0.03, *V* = 0.18); men exhibited slightly more positive attitudes about sharing information. No significant differences were found based on age groups (χ^2^ (6) = 7.14, *p* = 0.31, *V* = 0.11).

### 3.4. Cross-Analysis between Attitudes towards Obtaining and Sharing Information

Considering the perceived helpfulness of user-shared health information regarding OTC medication as an independent variable, a cross-analysis was conducted with the use of the health-related applications and anonymized sharing of health information as dependent variables. Respondents were divided into “relatively high perceived helpfulness” and “relatively low perceived helpfulness” based on their attitudes.

First, as Table 5 shows, regardless of the attitude towards user-shared health information, most participants owned smartphones but used neither application. The results comparing relatively high and low perceived helpfulness indicated that health-related application usage was slightly higher for those who found user-shared health information helpful (χ^2^ (4) = 18.35, *p* = 0.001, *V* = 0.25).

As Table 6 shows, the results comparing relatively high and low perceived helpfulness implied that the higher the perceived helpfulness of user-shared health information, the higher the proportion of positive attitudes towards anonymized sharing of health information (χ^2^ (3) = 19.78, *p* < 0.001, *V* = 0.26).

## 4. Discussion

This study tested the hypotheses for the OTC-EHR conceptual design from the perspectives of obtaining and sharing anonymized health information regarding OTC medication usage for self-medication.

Participants, especially women and those with high eHealth literacy indicated relatively positive attitudes towards the use of user-shared OTC medication information, thus partially supporting hypotheses H1 and H3. This phenomenon may be explained by the fact that high eHealth literacy consumers are better at obtaining and judging medical and health information from digital sources [8,10,40], and the perceived utility of health information technology tools is correlated with health literacy levels [22]. The higher motivation of women in this study may be because they are more likely to seek health information [43,44,63].

Combining the quantitative and text-mining analysis results, most consumers were concerned about the OTC medication information, including efficacy, safety, side effects, and persistence. Furthermore, regarding the user-shared medication information, a portion of consumers with high eHealth literacy, women, and consumers in the older age groups were inclined to know more medication-related details, such as the physical condition of the person reporting the medication information, their real experiences, and whether they have been to the hospital, to help their self-decision. These differences in information needs may be influenced by experience, information accessibility, and different medication needs [1,40,44], and more evidence is needed to explain these phenomena.

Hence, it is necessary to design the OTC medication information through an appropriate interface, including personalized detailed medication information of those who shared their information, to reach different users with different characteristics more efficiently and comprehensively, while improving their potential barriers and risks in accessing medical information. In addition, reducing the potential digital divide caused by different consumer characteristics and ensuring equal access to information on OTC-EHR through design must be addressed, which requires more studies [27].

In terms of the results of the attitudes towards sharing anonymized health information regarding OTC medication, the proportion of participants who use health applications is low; most consumers only have smartphones and do not use applications to record and report their health information. The study findings did not indicate that consumers with higher eHealth literacy have a more positive attitude towards medication information-sharing. Thus, hypotheses H2 and H4 were not supported. This potential resistance to anonymized health information sharing may emerge from, on the one hand, consumers’ concerns about information security and privacy [34,39,64]. Even though we clarified to the participants that the information shared would be anonymized, it did not reduce their psychological barriers. On the other hand, resistance may also be because of the penetration rate of EHRs [27,35], and the low usage rate of health-related applications found in this study.

It is noteworthy, however, that consumers’ favorable attitudes towards technology may facilitate greater use, as the perceived usefulness of EHR may have a positive effect on EHR use [27,36]. Our results also suggest that the perceived helpfulness of OTC-EHR by participants in this study may be positively associated with the usage of health-related applications and attitude towards sharing anonymized health information. Hypothesis H5 is partially confirmed. Therefore, first, it is important to use appropriate information and interface design to emphasize information security to reduce users’ psychological barriers. Second, the penetration rate of OTC-EHR should be improved to enhance the limited motivation to share anonymized health information regarding OTC medication. Considering the positive role of personal feedback on EHR [65], conducting OTC-EHR using a small database and encouraging consumers to have more access to OTC-EHR so as to improve their attitude towards information sharing is recommended. In addition, regarding OTC-EHR as a database of medication usage, maintaining design consistency and developing uniform design standards may positively reduce barriers for consumers in various usage scenarios, reduce user confusion, and potentially improve the use of OTC-EHR.

### Limitations

The present study has some limitations. First, this study did not explain in detail how agencies review relevant information, which will be studied in further research. Second, due to the limitations of research methods, this study did not explain in detail the interaction between the motivation to obtain and share anonymized health information regarding OTC medication, particularly the reasons for the psychological barriers to sharing anonymized health information. Considering the penetration rate of EHRs and health-related applications, this needs to be studied soon in conjunction with prototyping. Experiments based on prototyping will improve the knowledge on user behavior and perception. Third, the study sample has a user recruitment bias, as age groups older than 49 years are not addressed in this study. There is a need for a separate study on EHRs and the behavior of the elderly, given Japan’s aging population [66] and the potential for digital behavior change [44]. In addition, the present study was only conducted in the Kanto area of Japan to reduce the potential influence of cultural differences. It also excluded the impact of professional knowledge as those in medicine-related occupations. Therefore, future studies will expand the research scope by addressing these issues.

## 5. Conclusions

This study suggests that Japanese consumers, particularly women and those with high eHealth literacy, possess relatively positive attitudes towards obtaining user-shared OTC medication information, rather than towards sharing anonymized health information. The OTC-EHR suggested in this study holds the potential to enhance consumers’ self-medication, while the psychological barriers to sharing anonymized health information should be improved by increasing the penetration rate of OTC-EHR and engaging in appropriate information design.

## Figures and Tables

**Table 1:** Perceived helpfulness of obtaining user-shared health information on OTC medication.

	Not Helpful at All	Not Very Helpful	Neither	Helpful	Very Helpful
eHealth literacy					
High J-eHEALS	0 (0.00%)	7 (5.04%)	41 (29.50%)	75 (54.96%)	16 (11.51%)
Low J-eHEALS	6 (4.03%)	18 (12.08%)	55 (36.91%)	62 (41.61%)	8 (5.37%)
Gender					
Women	1 (0.67%)	7 (4.67%)	50 (33.33%)	78 (52.00%)	14 (9.33%)
Men	5 (3.62%)	18 (13.04)	46 (33.33%)	59 (42.75%)	10 (7.25%)
Age groups					
20–29	5 (5.26%)	9 (9.47%)	25 (26.32%)	46 (48.42%)	10 (10.53%)
30–39	1 (1.14%)	8 (9.09%)	32 (36.36%)	38 (43.18%)	9 (10.23%)
40–49	0 (0.00%)	8 (7.62%)	39 (37.14%)	53 (50.43%)	5 (4.76%)
Total	6 (2.08%)	25 (8.68%)	96 (33.33%)	137 (47.57%)	24 (8.33%)

**Table 2:** Information that respondents wished to obtain from the user report.

	Efficacy	Safety	Side Effects	Time to Produce the Effect	Duration of the Medication	Side Effect Incidence Rate	I Do Not Want to Know any Information	Others
eHealth literacy								
High J-eHEALS	95 (68.35%)	95 (68.35%)	90 (64.75%)	71 (51.08%)	64 (46.04%)	61 (43.88%)	14 (10.07%)	2 (1.44%)
Low J-eHEALS	93 (62.42%)	90 (60.40%)	86 (57.72%)	69 (46.31%)	56 (37.58%)	50 (33.56%)	25 (16.78%)	0 (0.00%)
Genders								
Women	98 (65.33%)	102 (68.00%)	109 (72.67%)	87 (58.00%)	80 (53.33%)	74 (49.33%)	18 (12.00%)	2 (1.33%)
Men	90 (65.22%)	83 (60.14%)	67 (48.55%)	53 (38.41%)	40 (28.99%)	37 (26.81%)	21 (15.22%)	0 (0.00%)
Age groups								
20–29	60 (63.16%)	61 (64.21)	51 (53.68%)	44 (46.32%)	42 (44.21%)	29 (30.53%)	12 (12.63%)	2 (2.11%)
30–39	58 (65.91%)	54 (61.36%)	55 (62.50%)	48 (54.55%)	34 (38.64%)	33 (37.50%)	11 (12.50%)	0 (0.00%)
40–49	70 (66.67%)	70 (66.67%)	70 (66.67%)	48 (45.71%)	44 (41.90%)	49 (46.67%)	16 (15.24%)	0 (0.00%)
Total	188 (65.28%)	185 (64.24%)	176 (61.11%)	140 (48.61%)	120 (41.67%)	111 (38.54%)	39 (13.54%)	2 (0.69%)

**Table 3:** The use of health-related applications on smartphones.

	Medication Notebook Application	Health Observation Application	Both Medication Notebook and Health Observation Applications	Have a Smartphone but Use Neither Application	I Do Not Use a Smartphone
eHealth literacy					
High J-eHEALS	13 (9.35%)	13 (9.35%)	8 (5.76%)	92 (66.19%)	13 (9.35%)
Low J-eHEALS	12 (8.05%)	4 (2.68%)	3 (2.01%)	108 (72.48%)	22 (14.77%)
Genders					
Women	14 (9.33%)	13 (8.67%)	7 (4.67%)	94 (62.67%)	22 (14.67%)
Men	11 (7.97%)	4 (2.90%)	4 (2.90%)	106 (76.81%)	13 (9.42%)
Age groups					
20–29	11 (11.58%)	7 (7.37%)	2 (2.11%)	63 (66.32%)	12 (12.63%)
30–39	8 (10.23%)	8 (10.23%)	3 (3.41%)	61 (69.32%)	8 (10.23%)
40–49	6 (5.71%)	2 (1.90%)	6 (5.71%)	76 (72.38%)	15 (14.29%)
Total	25 (8.68%)	17 (5.90%)	11 (3.82%)	200 (69.44%)	35 (12.15%)

**Table 4:** Attitudes towards sharing anonymized health information.

	I Think It Is a Good Thing	Not Okay	Neither	I Do Not Know
eHealth literacy				
High J-eHEALS	33 (29.73%)	30 (27.03%)	40 (36.04%)	8 (7.21%)
Low J-eHEALS	39 (22.03%)	38 (21.47%)	67 (37.85%)	33 (18.64%)
Gender				
Women	35 (23.33%)	43 (28.67%)	58 (38.67%)	14 (9.33%)
Men	37 (26.81%)	25 (18.12%)	49 (35.51%)	27 (19.57%)
Age groups				
20–29	31 (32.63%)	16 (16.84%)	33 (34.74%)	15 (15.79%)
30–39	20 (22.73%)	22 (25.00%)	33 (37.50%)	13 (14.77%)
40–49	21 (20.00%)	30 (28.57%)	41 (39.05%)	13 (12.38%)
Total	72 (25.00%)	68 (23.61%)	107 (37.15%)	41 (14.24%)

**Table 5:** Cross-analysis between perceived helpfulness of user-shared information and health-related application use.

	Medication Notebook Application	Health Observation Application	Both Medication Notebook and Health Observation Applications	Have a Smartphone but Use Neither Application	I Do Not Use a Smartphone
Very helpful	3 (12.50%)	2 (8.33%)	0 (0.00%)	17 (70.83%)	2 (8.33%)
Helpful	12 (8.76%)	13 (9.49%)	7 (5.11%)	97 (70.80%)	8 (5.84%)
^1^ Relatively high perceived helpfulness	15 (9.32%)	15 (9.32%)	7 (4.35%)	114 (70.81%)	10 (6.21%)
Neither	6 (6.25%)	1 (1.04%)	4 (4.17%)	66 (68.75%)	19 (19.79)
Not very helpful	2 (8.00%)	1 (4.00%)	0 (0.00%)	16 (64.00%)	6 (24.00%)
Not helpful at all	2 (33.33%)	0 (0.00%)	0 (0.00%)	4 (66.67%)	0 (0.00%)
^2^ Relatively low perceived helpfulness	10 (7.87%)	2 (1.57%)	4 (3.15%)	86 (67.72%)	25 (19.69%)

^1^ Participants who chose “very helpful” and “helpful”. ^2^ Participants who chose “neither”, “not very helpful”, and “not helpful at all”.

**Table 6:** Cross-analysis between user-reported information perceived helpfulness and anonymized sharing of health information.

	I Think It Is a Good Thing	Not Okay	Neither	I Do Not Know
Very helpful	13 (54.17%)	6 (25.00%)	3 (12.50%)	2 (8.33%)
Helpful	39 (28.47%)	38 (27.74%)	47 (34.31%)	13 (9.49%)
^1^ Relatively high perceived helpfulness	52 (32.30%)	44 (27.33%)	50 (31.06%)	15 (9.32%)
Neither	12 (12.50%)	17 (17.71%)	49 (51.04%)	18 (18.75%)
Not very helpful	6 (24.00%)	6 (24.00%)	7 (28.00%)	6 (24.00%)
Not helpful at all	2 (33.33%)	1 (16.67%)	1 (16.67%)	2 (33.33%)
^2^ Relatively low perceived helpfulness	20 (15.75%)	24 (18.90%)	57 (44.88%)	26 (20.47%)

^1^ Participants who chose “very helpful” and “helpful”. ^2^ Participants who chose “neither”, “not very helpful”, and “not helpful at all”.

## Data Availability

The data that support the findings of this study are available from the corresponding authors upon reasonable request.

## Supplementary Materials

The following supporting information can be downloaded at: https://www.mdpi.com/article/10.3390/healthcare11081166/s1, Table S1: Survey questions and hypotheses.
