# Supplementary material for: Japanese Consumers’ Attitudes towards Obtaining and Sharing Health Information Regarding Over-the-Counter Medication: Designing an Over-the-Counter Electronic Health Record
Source: Healthcare (Basel). 2023 Apr 18;11(8):1166. doi: 10.3390/healthcare11081166 (PMC10138617; doi:10.3390/healthcare11081166)
Supplement: Supplementary file 1 [file healthcare-11-01166-s001.zip › healthcare-2300947-supplementary.pdf]

Table S1. Survey questions and hypotheses

Research Hypotheses
H1: Consumers exhibit a positive attitude towards obtaining user-shared health information on OTC medication.<br><br>H2: Consumers exhibit a positive attitude towards sharing anonymized health information while using OTC medication.<br><br>H3: There is a positive association between consumers obtaining and sharing health information regarding OTC medication.<br><br>H4: Consumers with high eHealth literacy have a greater inclination to obtain user-shared information regarding OTC medication.<br><br>H5: Consumers with high eHealth literacy are more optimistic about sharing anonymized health information regarding OTC medication.

Survey Questions	Corresponding Research Hypotheses
**Participant characteristics**	
Japanese version of the eHealth Literacy Scale (J-eHEALS) [51]	H4, H5
Gender	
Age	
**Attitudes towards obtaining user-shared OTC medication information**	
(i) If there were a database of post-dose health information shared from past users of the OTC medication you purchase, do you think it would be helpful when deciding to choose the medication?<br>The response options ranged from “very helpful = 5” to “not helpful at all = 1.”	H1, H3, H4
(ii) What information would you like to refer to in this user-shared report?<br>Respondents were asked to select all suitable answers from the following: “efficacy of medicines,” “safety of medicines,” “average time from taking a medicine to effects,” “duration of drug effect,” “symptoms of side effects,” “incidence of side effects,” “others,” “I do not want to know any information.”	H1
(iii) Enter the reasons for choosing the responses in the free response text box.	H1
**Usage of health-related applications and inclination to share anonymized health information**	
(i) Do you use a medication notebook application or an application that monitors your health condition, such as entering body temperature, on your smartphone?<br>Respondents were asked to select one of the following answers that best applies to them: “I only use the medication notebook application,” “I only use the health observation application,” “I use both the medication notebook and the health observation applications,” “I have a smartphone but use neither application,” and “I do not use a smartphone.”	H3
(ii) Do you think it is acceptable for information about your physical condition and medicines used entered into the application to be provided to others after anonymization?<br>The answers were “I think it is a good thing,” “not okay,” “neither,” and “I do not know.”	H2, H3, H5
